# Correction: Maternal mortality associated with COVID-19 in Brazil in 2020 and 2021: Comparison with non-pregnant women and men

**DOI:** 10.1371/journal.pone.0294703

**Published:** 2023-11-15

**Authors:** Beatriz Martinelli Menezes Gonçalves, Rossana Pulcinelli V. Franco, Agatha S. Rodrigues

The second author’s name is spelled incorrectly. The correct name is: Rossana Pulcinelli V. Francisco

The correct citation is: Gonçalves BMM, Francisco RPV, Rodrigues AS (2021) Maternal mortality associated with COVID-19 in Brazil in 2020 and 2021: Comparison with non-pregnant women and men. PloS ONE 16(12): e0261492. https://doi.org/10.1371/journal.pone.0261492
