# Supplementary material for: Maternal mortality associated with COVID-19 in Brazil in 2020 and 2021: Comparison with non-pregnant women and men
Source: PLoS One. 2021 Dec 21;16(12):e0261492. doi: 10.1371/journal.pone.0261492 (PMC8691656; doi:10.1371/journal.pone.0261492)
Supplement: Attachment — Submitted filename: Response to Reviewers.docx [file pone.0261492.s001.docx]

**Responses to the Reviewers and Editor:**

Dear Editor Dong Keon Yon

**1. #1. Documenting that pregnant and postpartum women are at a higher risk of developing severe forms of COVID-19 is essential for prioritizing them in health care strategies.**

**Please cite references.**

Response: Thank you for your comment. The following text has been included in the current version of the manuscript in the discussion section.

“Pregnant and postpartum women have been shown to be at a higher risk of developing more severe illnesses related to the SARS-COV-2 infection as highlighted by Zambrano et al. in November 2020 [11]. This data was further corroborated by a systematic review published by Wastnedge et al, also suggesting a higher vulnerability of the pregnant population to the infection [27]. Other studies also validateours, suggesting that pregnant women are at a higher risk for ICU care during the 2021 pandemic. [13,16].”

References:

11. Zambrano LD, Ellington S, Strid P, Galang RR, Oduyebo T, Tong VT, et al. Update: Characteristics of Symptomatic Women of Reproductive Age with Laboratory-Confirmed SARS-CoV-2 Infection by Pregnancy Status - United States, January 22-October 3, 2020. MMWR Morb Mortal Wkly Rep. 2020;69(44):1641-1647.
27. Wastnedge EAN, Reynolds RM, van Boeckel SR, Stock SJ, Denison FC, Maybin JA, Critchley HOD. Pregnancy and COVID-19. Physiol Rev. 2021 Jan 1;101(1):303-318. doi: 10.1152/physrev.00024.2020. Epub 2020 Sep 24. PMID: 32969772; PMCID: PMC7686875.
13. Nakamura-Pereira M, Andreucci CB, Menezes M., Knobel R. et al. Worldwide maternal deaths due to COVID-19: A brief review. Gynecology & Obstetrics. 2020; 152: 148-150.
16. Takemoto MLS, Menezes M de O de O, Andreucci CB, et al. Clinical characteristics and risk factors for mortality in obstetric patients with severe COVID-19 in Brazil: a surveillance database analysis. BJOG 2020; 127: 1618–26.

**2. Please explain plausible basic mechanism. (i.e., ACE2, SARS-CoV-2, pregnancy, molecular mechanism)**

Response:Thank you for your suggestion. The following text has been included in the current version of the manuscript.

Recent studies suggest that the expression of angiotensin converting enzyme 2 (ACE 2), a receptor used by SARS-CoV-2 in human placentas, could increase maternal population susceptibility to infection. [1] ACE 2 receptors actively participate in necessary hemodynamic adaptations through pregnancy, when consumed by SARS-CoV-2 this could increase risk for placental dysfunction increasing risk of worst outcomes after infection [29,30].

References :

29. Nobrega Cruz N. A., Stoll D., Casarini D. E., Bertagnolli M. Role of ACE2 in pregnancy and potential implications for COVID-19 susceptibility. ClinSci (Lond) 13 August 2021; 135 (15): 1805–1824. doi: <https://doi.org/10.1042/CS20210284>

30. Todros T, Masturzo B, De Francia S. COVID-19 infection: ACE2, pregnancy and preeclampsia. Eur J ObstetGynecolReprod Biol. 2020;253:330. doi:10.1016/j.ejogrb.2020.08.007

**3. The authors have to provide the policy implication regarding vaccination. Please see the landmark previos study.**

**Hwang J, Park SH, Lee SW, Lee SB, Lee MH, Jeong GH, Kim MS, Kim JY, Koyanagi A, Jacob L, Jung SY, Song J, Yon DK, Shin JI, Smith L. Predictors of mortality in thrombotic thrombocytopenia after adenoviral COVID-19 vaccination: the FAPIC score. Eur Heart J. 2021 Sep 21:ehab592. doi: 10.1093/eurheartj/ehab592. Epub ahead of print. PMID: 34545400; PMCID: PMC8500026.**

Thank you for your comment. We did not provide any information regarding vaccination policy for pregnant and postpartum women because the studied period is previous to the maternal population vaccination start. The vaccination in Brazil for maternal population started in May 2021.

The following text has been included in the current version of the manuscript (Discussion):

“The period studied is prior to the start of vaccination of the maternal population. Vaccination in Brazil for the maternal population began in May 2021. Our study confirms the worse evolution of the population of pregnant and postpartum women when compared to non-pregnant women and men and may be useful in guiding this population regarding the importance of vaccination against COVID-19. Most references point out that the probability of complications arising during pregnancy due to SARS-COV2 infection is greater than those related to vaccination [31,32]. Currently, the maternal and postpartum population is considered at high risk for SARS-COV2 infection and should be among the priorities for vaccination.”

31. Rasmussen et al. COVID-19 Vaccines and Pregnancy. American College of Obstetricians and Gynecologists. 2021; 137 (3).

32. Russell, F. M. and Greenwood, B. Who should be prioritised for COVID-19 vaccination? Human Vaccin Immunother. 2021; 17(3): 1317-1321.

**4. Pregnancy patients was associted with depression [1] and low level physical activity [2], which may mediated the COVID-19 effect.**

**Please disscuss this in the limitation section and cite the top-tier papers below.**

Thank you for your suggestion. The following remarks were added to our discussion into the limitation section.

“The presence of health disorders and the role of physical activity were not analyzed in the present study. Increased mortality in patients with mental disorders has been described by Fond et al. in previous studies, OR (1.38 [95% CI, 1.15-1.65]; P < .05). [33] Physical activity was also identified as a protective factor, both for aerobic and muscular strengthening activities (0.02% vs 0.08%; aRR 0.24; 95% CI 0.05 to 0.99) [34]”

33. Fond G, Nemani K, Etchecopar-Etchart D, Loundou A, Goff DC, Lee SW, Lancon C, Auquier P, Baumstarck K, Llorca PM, Yon DK, Boyer L. Association Between Mental Health Disorders and Mortality Among Patients With COVID-19 in 7 Countries: A Systematic Review and Meta-analysis. JAMA Psychiatry. 2021 Jul 27:e212274. doi: 10.1001/jamapsychiatry.2021.2274. Epub ahead of print. PMID: 34313711; PMCID: PMC8317055.

34. Lee SW, Lee J, Moon SY, Jin HY, Yang JM, Ogino S, Song M, Hong SH, AbouGhayda R, Kronbichler A, Koyanagi A, Jacob L, Dragioti E, Smith L, Giovannucci E, Lee IM, Lee DH, Lee KH, Shin YH, Kim SY, Kim MS, Won HH, Ekelund U, Shin JI, Yon DK. Physical activity and the risk of SARS-CoV-2 infection, severe COVID-19 illness and COVID-19 related mortality in South Korea: a nationwide cohort study. Br J Sports Med. 2021 Jul 22:bjsports-2021-104203. doi: 10.1136/bjsports-2021-104203. Epub ahead of print. PMID: 34301715; PMCID: PMC8300550.

**Reviewer #2:**

**I may need more introduction to understand the problem in the region of Brazil.**

Thank you for your comment. The following text was added to our paper.

“In early 2021, the maternal mortality rate seems to be even higher, even though the knowledge about the disease and the treatment tools have evolved. A recent study by Takemoto *et. al,* demonstrated that the mortality rate was about two times higher for the maternal population in 2021 (15.6%), when compared to 2020 (7.4%), but these authors did not compare the group of pregnant and postpartum women with the groups of men and women , as proposed in this study (7.4%) [15,16]. “

15. Takemoto MLS, Menezes M de O de O, Andreucci CB, et al. Higher case fatality rate among obstetric patients with COVID-19 in the second year of pandemic in Brazil: do new genetic variants play a role? medRxiv doi:https://doi.org/10.1101/2021.05.06.21256651.

16. Takemoto MLS, Menezes M de O de O, Andreucci CB, et al. Clinical characteristics and risk factors for mortality in obstetric patients with severe COVID-19 in Brazil: a surveillance database analysis. BJOG 2020; 127: 1618–26.
